# Supplementary material for: COVID-19 Vaccine Concerns about Safety, Effectiveness, and Policies in the United States, Canada, Sweden, and Italy among Unvaccinated Individuals
Source: Vaccines (Basel). 2021 Oct 6;9(10):1138. doi: 10.3390/vaccines9101138 (PMC8538553; doi:10.3390/vaccines9101138)
Supplement: Supplementary file 1 [file vaccines-09-01138-s001.zip › vaccines-1395511-supplementary.pdf]

Supplementary Material – Confirmatory Factor Analysis

Item	*Factor Loading*
Vaccine Safety and Government Control – Factor 1	
1. You can get COVID-19 from the vaccine itself (R)	0.6668
2. There are toxic ingredients in the vaccine that can harm your health (R)	0.7663
3. The vaccine can mess up your DNA (R)	0.7268
4. The vaccine can cause infertility (R)	0.7359
5. The vaccine can cause other diseases (R)	0.7356
6. The fast production of the vaccine compromised its safety (R)	0.6907
7. The vaccine is going to be used by Governments as a tool to limit our civil rights (right of assembly, right of movement, right of religion, etc.)	0.6808

Item	*Factor Loading*
Vaccine Effectiveness and Population Control – Factor 1	
8. The vaccine will work in protecting me from getting COVID-19	0.7406
9. By taking the vaccine I will protect my friends and family from getting COVID-19	0.7926
10. Everyone should get the vaccine to achieve herd immunity	0.8098
11. Getting COVID-19 is worse than experiencing potential side effects from the vaccine	0.6863
12. Natural remedies will not protect me from COVID-19	0.6385

Item	*Factor Loading*	
	1	***2***
Freedom – Factor 1		
13. People should be free to decide if they get vaccinated or not with no consequences for their job or personal life	0.807	
14. People should have the option to choose the vaccine brand they want	0.7607	
15. People should be allowed to live their life with no restrictions once vaccinated	0.437	
16. Healthcare professionals and scientists with concerns about the vaccine should have opportunities to share their opinions with the public	0.6284	
**Freedom – Factor 2**		
17. Everybody should have equal access to the most effective and safe vaccine regardless of income, race, or immigration status		0.8364
18. There is no elite group that will achieve financial power if people are getting vaccinated		0.6319
19. There is no microchip with tracking capabilities inserted in the vaccine		0.7415

Supplementary Material – Exploratory Factor Analysis

Item	*Factor Loading*		
	1	*2*	*3*
Vaccine Safety and Government Control – Factor 1			
1. You can get COVID-19 from the vaccine itself (R)	0.6186		
2. There are toxic ingredients in the vaccine that can harm your health (R)	0.7085		
3. The vaccine can mess up your DNA (R)	0.609		
4. The vaccine can cause infertility (R)	0.6747		
5. The vaccine can cause other diseases (R)	0.7362		
6. The fast production of the vaccine compromised its safety (R)	0.6288		
7. The vaccine is going to be used by Governments as a tool to limit our civil rights (right of assembly, right of movement, right of religion, etc.)	0.5471		
Vaccine Effectiveness and Population Control – Factor 2			
8. The vaccine will work in protecting me from getting COVID-19		0.7406	
9. By taking the vaccine I will protect my friends and family from getting COVID-19		0.7926	
10. Everyone should get the vaccine to achieve herd immunity		0.8098	
11. Getting COVID-19 is worse than experiencing potential side effects from the vaccine		0.6863	
12. Natural remedies will not protect me from COVID-19		0.6385	
18. There is no elite group that will achieve financial power if people are getting vaccinated		0.4253	
19. There is no microchip with tracking capabilities inserted in the vaccine		0.6399	
17. Everybody should have equal access to the most effective and safe vaccine regardless of income, race, or immigration status		0.5879	0.4606
Freedom – Factor 3			
13. People should be free to decide if they get vaccinated or not with no consequences for their job or personal life			0.7292
14. People should have the option to choose the vaccine brand they want			0.7259
15. People should be allowed to live their life with no restrictions once vaccinated			0.5287
16. Healthcare professionals and scientists with concerns about the vaccine should have opportunities to share their opinions with the public			0.7029

Supplementary Material – Mean Agreement and Standard Deviation for All Items

	Reason	Overall Sample	United States			Canada			Sweden			Italy		
			Overall	Non-Hesitant ^	Hesitant	Overall	Non-Hesitant	Hesitant	Overall	Non-Hesitant	Hesitant	Overall	Non-Hesitant	Hesitant
**Vaccine Safety and Government Control**	1. You can get COVID-19 from the vaccine itself (R) *, +, -	4.9 (2.8)	5.3 (2.9)	**4.1 (3)**	6 (2.7)	4.2 (2.7)	3.3 (2.5)	5.5 (2.4)	5 (2.7)	4.4 (2.8)	5.8 (2.4)	**5.3 (2.8)**	**4.9 (2.9)**	5.8 (2.6)
	2. There are toxic ingredients in the vaccine that can harm your health (R) *, +, -	5.1 (2.6)	5.3 (2.9)	4 (2.9)	6.1 (2.7)	4.8 (2.6)	3.9 (2.4)	6.1 (2.4)	5.5 (2.4)	4.8 (2.3)	**6.3 (2.1)**	4.7 (2.5)	4 (2.3)	5.6 (2.5)
	3. The vaccine can mess up your DNA (R) *, +, -	4.4 (2.8)	5 (2.9)	3.7 (2.9)	5.8 (2.7)	4.2 (2.7)	3.2 (2.4)	5.4 (2.5)	4.5 (2.8)	3.8 (2.9)	5.2 (2.4)	4.2 (2.6)	3.6 (2.6)	4.9 (2.5)
	4. The vaccine can cause infertility (R) *, +, -	4.8 (2.6)	5.3 (2.8)	3.9 (2.8)	6.1 (2.5)	4.5 (2.6)	3.6 (2.4)	5.7 (2.3)	5.1 (2.4)	4.6 (2.5)	5.7 (2.2)	4.6 (2.6)	4.1 (2.7)	5.1 (2.3)
	5. The vaccine can cause other diseases (R) *, -	**5.3 (2.6)**	5.3 (2.9)	3.8 (2.9)	6.2 (2.6)	4.8 (2.6)	4 (2.5)	6.1 (2.3)	**5.8 (2.3)**	**5.3 (2.3)**	6.3 (2.2)	5.2 (2.5)	4.6 (2.5)	**5.9 (2.5)**
	6. The fast production of the vaccine compromised its safety (R) *, +, -	5.2 (2.7)	**5.6 (3)**	4 (3)	**6.5 (2.7)**	**5 (2.6)**	**4.1 (2.4)**	**6.3 (2.4)**	5.5 (2.5)	5 (2.4)	6.1 (2.4)	4.9 (2.7)	4.3 (2.5)	5.7 (2.6)
	7. The vaccine is going to be used by Governments as a tool to limit our civil rights (right of assembly, right of movement, right of religion, etc.) (R) *, +	4.9 (2.9)	5.4 (3.1)	3.9 (2.9)	6.2 (2.8)	4.8 (2.9)	3.9 (2.7)	6 (2.6)	4.9 (2.7)	4.1 (2.8)	5.8 (2.4)	4.5 (2.8)	4 (2.7)	5.2 (2.8)
**Vaccine Effectiveness and Population Control**	8. The vaccine will work in protecting me from getting COVID-19 *, +, -	6.8 (2.7)	6 (3.1)	7.7 (2.7)	5 (2.8)	7.2 (2.6)	8.3 (2)	5.6 (2.6)	6.6 (2.6)	7.8 (2.1)	5.4 (2.5)	7.3 (2.4)	8.2 (1.8)	6 (2.4)
	9. By taking the vaccine I will protect my friends and family from getting COVID-19 *, +, -	7.1 (2.8)	6.1 (3)	7.8 (2.7)	5.1 (2.8)	7.4 (2.7)	8.6 (2)	5.7 (2.6)	7 (2.8)	8.4 (2.2)	5.5 (2.6)	7.8 (2.4)	8.7 (1.9)	6.5 (2.5)
	10. Everyone should get the vaccine to achieve herd immunity *, +, -	7.1 (2.9)	**5.8 (3.1)**	7.6 (2.7)	**4.7 (2.8)**	7.4 (2.8)	8.8 (1.8)	5.5 (2.8)	7.1 (2.7)	8.7 (1.9)	5.5 (2.5)	7.7 (2.6)	8.8 (1.9)	6.3 (2.6)
	11. Getting COVID-19 is worse than experiencing potential side effects from the vaccine *, -	6.7 (2.8)	5.9 (2.9)	**7 (3.1)**	5.3 (2.6)	7 (2.8)	8.1 (2.5)	5.5 (2.6)	6.7 (2.7)	7.9 (2.5)	**5.4 (2.3)**	6.9 (2.7)	7.9 (2.4)	5.6 (2.4)
	12. Natural remedies will not protect me from COVID-19 *, +, -	6.9 (2.8)	6 (2.9)	7.2 (2.9)	5.3 (2.6)	7.1 (2.8)	7.9 (2.6)	5.9 (2.6)	6.9 (2.7)	7.8 (2.7)	5.9 (2.5)	7.4 (2.6)	8.1 (2.4)	6.6 (2.4)
	17. Everybody should have equal access to the most effective and safe vaccine regardless of income, race, or immigration status *, +, -	8 (2.3)	7 (2.5)	7.3 (2.5)	6.8 (2.4)	8 (2.3)	8.7 (1.8)	7.1 (2.6)	8.1 (2.4)	8.9 (1.9)	7.3 (2.6)	8.6 (2)	9 (1.8)	8 (2.1)
	18. There is no elite group that will achieve financial power if people are getting vaccinated *, -	**6.1 (2.7)**	5.9 (2.7)	7 (2.5)	5.2 (2.6)	**6.1 (2.7)**	**6.7 (2.6)**	**5.3 (2.7)**	**6.6 (2.7)**	**7.5 (2.5)**	5.5 (2.5)	**6 (2.7)**	**6.5 (2.7)**	**5.3 (2.6)**
	19. There is no microchip with tracking capabilities inserted in the vaccine *, +, -	7.3 (2.6)	6.2 (2.6)	7.2 (2.4)	5.6 (2.5)	7.5 (2.5)	8.3 (2.1)	6.4 (2.6)	7.3 (2.7)	8.4 (2.4)	6.2 (2.6)	7.9 (2.4)	8.6 (2.1)	7.1 (2.4)
**Freedom**	13. People should be free to decide if they get vaccinated or not with no consequences for their job or personal life *, +, -	**6.3 (2.8)**	6.8 (2.6)	**6.8 (2.6)**	6.9 (2.5)	**6.1 (2.9)**	**5.4 (2.9)**	6.9 (2.7)	**6.4 (2.8)**	**6 (2.9)**	6.9 (2.6)	**6.2 (2.9)**	**5.2 (2.9)**	7.4 (2.5)
	14. People should have the option to choose the vaccine brand they want *, +, -	7.1 (2.5)	7.2 (2.3)	7.5 (2.3)	7 (2.3)	7.1 (2.4)	7.3 (2.2)	6.9 (2.6)	6.8 (2.7)	6.6 (2.6)	7 (2.7)	7.3 (2.6)	7 (2.8)	7.6 (2.3)
	15. People should be allowed to live their life with no restrictions once vaccinated *, +, -	6.7 (2.5)	**6.6 (2.5)**	7.1 (2.6)	**6.3 (2.4)**	6.5 (2.6)	6.7 (2.5)	**6.3 (2.6)**	6.5 (2.5)	6.9 (2.3)	**6.1 (2.5)**	7.2 (2.4)	7.5 (2.3)	**7 (2.5)**
	16. Healthcare professionals and scientists with concerns about the vaccine should have opportunities to share their opinions with the public *, +, -	7.2 (2.4)	6.9 (2.4)	7.2 (2.4)	6.8 (2.4)	7.4 (2.3)	7.6 (2.1)	7.1 (2.4)	7.2 (2.4)	7.6 (2.2)	6.9 (2.5)	7.1 (2.5)	6.9 (2.6)	7.5 (2.4)

Key:
** KW test by country is significant for overall sample*
+ KW test by country is significant for hesitant sample
- KW test by country is significant for non-hesitant sample
^ Repeated measures model did not indicate that the means for Vaccine Safety and Government Control were significantly different among this sub-sample

Supplementary Material – Additional Psychometric Analyses

Correlation Matrix of Items by Subscale

Item	1	2	3	4	5	6	7	8	9	10	11	12	17	18	19	13	14	15	16
1. You can get COVID-19 from the vaccine itself (R)	–																		
2. There are toxic ingredients in the vaccine that can harm your health (R)	0.44*	–																	
3. The vaccine can mess up your DNA (R)	0.41*	0.46*	–																
4. The vaccine can cause infertility (R)	0.42*	0.48*	0.48*	–															
5. The vaccine can cause other diseases (R)	0.41*	0.52*	0.43*	0.46*	–														
6. The fast production of the vaccine compromised its safety (R)	0.35*	0.47*	0.4*	0.42*	0.43*	–													
7. The vaccine is going to be used by Governments as a tool to limit our civil rights (right of assembly, right of movement, right of religion, etc.)	0.35*	0.43*	0.44*	0.4*	0.4*	0.4*	–												
8. The vaccine will work in protecting me from getting COVID-19	0.41*	0.46*	0.43*	0.41*	0.41*	0.41*	0.41*	–											
9. By taking the vaccine I will protect my friends and family from getting COVID-19	0.37*	0.44*	0.43*	0.41*	0.38*	0.39*	0.42*	0.7*	–										
10. Everyone should get the vaccine to achieve herd immunity	0.36*	0.46*	0.45*	0.42*	0.39*	0.42*	0.43*	0.68*	0.72*	–									
11. Getting COVID-19 is worse than experiencing potential side effects from the vaccine	0.34*	0.38*	0.38*	0.37*	0.35*	0.36*	0.36*	0.53*	0.54*	0.58*	–								
12. Natural remedies will not protect me from COVID-19	0.31*	0.34*	0.38*	0.34*	0.3*	0.32*	0.36*	0.46*	0.47*	0.49*	0.45*	–							
17. Everybody should have equal access to the most effective and safe vaccine regardless of income, race, or immigration status	0.22*	0.18*	0.28*	0.26*	0.16*	0.19*	0.26*	0.35*	0.42*	0.4*	0.35*	0.36*	–						
18. There is no elite group that will achieve financial power if people are getting vaccinated	0.29*	0.33*	0.34*	0.3*	0.29*	0.3*	0.34*	0.38*	0.37*	0.41*	0.36*	0.31*	0.25*	–					
19. There is no microchip with tracking capabilities inserted in the vaccine	0.35*	0.36*	0.43*	0.38*	0.3*	0.29*	0.36*	0.48*	0.49*	0.5*	0.44*	0.45*	0.44*	0.36*	–				
13. People should be free to decide if they get vaccinated or not with no consequences for their job or personal life	-0.06*	-0.14*	-0.08*	-0.06*	-0.08*	-0.11*	-0.12*	-0.14*	-0.13*	-0.2*	-0.11*	-0.08*	0.11*	-0.01*	-0.04*	–			
14. People should have the option to choose the vaccine brand they want	0.02	-0.02	0.03	0.02	-0.01	-0.01	0.01	0.05*	0.06*	0.03*	0.05*	0.09*	0.3*	0.03	0.12*	0.36*	–		
15. People should be allowed to live their life with no restrictions once vaccinated	0.15*	0.15*	0.15*	0.13*	0.13*	0.12*	0.1*	0.23*	0.23*	0.2*	0.18*	0.18*	0.27*	0.17	0.23*	0.22*	0.28*	–	
16. Healthcare professionals and scientists with concerns about the vaccine should have opportunities to share their opinions with the public	0.09*	0.01	0.1*	0.09*	0.05*	0.02	0.07*	0.11*	0.16*	0.12*	0.1*	0.14*	0.38*	0.13*	0.23*	0.33*	0.37*	0.25*	–

*$p < 0.05$

Correlations Between Items and Subscale Score

Item	Subscale		
	Safety	Effectiveness	Freedom
1. You can get COVID-19 from the vaccine itself (R)	0.68		
2. There are toxic ingredients in the vaccine that can harm your health (R)	0.76		
3. The vaccine can mess up your DNA (R)	0.73		
4. The vaccine can cause infertility (R)	0.73		
5. The vaccine can cause other diseases (R)	0.73		
6. The fast production of the vaccine compromised its safety (R)	0.69		
7. The vaccine is going to be used by Governments as a tool to limit our civil rights (right of assembly, right of movement, right of religion, etc.)	0.69		

8. The vaccine will work in protecting me from getting COVID-19	0.80	
9. By taking the vaccine I will protect my friends and family from getting COVID-19	0.82	
10. Everyone should get the vaccine to achieve herd immunity	0.83	
11. Getting COVID-19 is worse than experiencing potential side effects from the vaccine	0.74	
12. Natural remedies will not protect me from COVID-19	0.69	
17. Everybody should have equal access to the most effective and safe vaccine regardless of income, race, or immigration status	0.60	
18. There is no elite group that will achieve financial power if people are getting vaccinated	0.60	
19. There is no microchip with tracking capabilities inserted in the vaccine	0.71	
13. People should be free to decide if they get vaccinated or not with no consequences for their job or personal life		0.72
14. People should have the option to choose the vaccine brand they want		0.72
15. People should be allowed to live their life with no restrictions once vaccinated		0.63
16. Healthcare professionals and scientists with concerns about the vaccine should have opportunities to share their opinions with the public		0.69

Cronbach Alpha for Each Subscale by Country

Subscale	Overall	US	CA	SWE	IT
Safety and Government Control (7 items)	0.84	0.89	0.88	0.81	0.73
Effectiveness and Population Control (8 items)	0.87	0.87	0.87	0.87	0.86
Freedom (4 items)	0.63	0.68	0.58	0.64	0.64

Supplementary Material – Sample Descriptive Statistics

Sex	US	%	CA	%	SWE	%	IT	%	Total	%
Male	364	50%	491	50%	477	49%	489	50%	1,821	50%
Female	363	50%	494	50%	488	51%	497	50%	1,842	50%
Total	727		985		965		986		3,663	

Education	US	%	CA	%	SWE	%	IT	%	Total	%
Less than high school	49	7%	49	5%	89	9%	83	8%	270	7%
High school or equivalent	162	22%	238	24%	463	48%	440	45%	1,303	36%
Some college	140	19%	276	28%	161	17%	133	13%	710	19%
Bachelor's degree	132	18%	296	30%	155	16%	277	28%	860	23%
Post-graduate degree	221	30%	118	12%	85	9%	51	5%	475	13%
Other	22	3%	8	1%	12	1%	2	0%	44	1%
Total	726		985		965		986		3,662	

Work Category	US	%	CA	%	SWE	%	IT	%	Total	%
Healthcare or social worker	29	4%	55	6%	75	8%	35	4%	194	5%
Residential facility	17	2%	3	0%	33	3%	11	1%	64	2%
Public health	7	1%	11	1%	11	1%	16	2%	45	1%
Correctional facility	4	1%	7	1%	5	1%	5	1%	21	1%
Vaccine manufacturing or distribution	3	0%	3	0%	5	1%	2	0%	13	0%
Pharmacy	7	1%	13	1%	6	1%	9	1%	35	1%
Teacher or school staff	24	3%	57	6%	63	7%	72	7%	216	6%
Food processing	10	1%	11	1%	11	1%	45	5%	77	2%
Grocery store	23	3%	49	5%	35	4%	37	4%	144	4%
Postal and shipping	5	1%	8	1%	15	2%	6	1%	34	1%
Transportation	15	2%	24	2%	57	6%	35	4%	131	4%
Police or firefighter	2	0%	8	1%	10	1%	24	2%	44	1%
Volunteer	37	5%	57	6%	23	2%	25	3%	142	4%
Other	250	34%	579	59%	558	58%	664	67%	2,051	56%
Multiple professions	294	40%	100	10%	58	6%	0	0%	452	12%
Total	727		985		965		986		3,663	

Supplementary Material – Survey Items

How much do you agree or disagree with the following statements?

	Strongly disagree	Disagree	Somewhat disagree	Unsure	Somewhat agree	Agree	Strongly agree
1. You cannot get COVID-19 from the vaccine itself							
2. There are no toxic ingredients in the vaccine that can harm your health							
3. The vaccine cannot mess up your DNA							
4. The vaccine cannot cause infertility							
5. The vaccine cannot cause other diseases							
6. The fast production of the vaccine did not compromise its safety							
7. The vaccine is not going to be used by Governments as a tool to limit our civil rights (right of assembly, right of movement, right of religion, etc.)							

How much do you agree or disagree with the following statements?

	Strongly disagree	Disagree	Somewhat disagree	Unsure	Somewhat agree	Agree	Strongly agree
8. The vaccine will work in protecting me from getting COVID-19							
9. By taking the vaccine I will protect my friends and family from getting COVID-19							
10. Everyone should get the vaccine to achieve herd immunity							
11. Getting COVID-19 is worse than experiencing potential side effects from the vaccine							
12. Natural remedies will not protect me from COVID-19							

How much do you agree or disagree with the following statements?

	Strongly disagree	Disagree	Somewhat disagree	Unsure	Somewhat agree	Agree	Strongly agree
13. People should be free to decide if they get vaccinated or not with no consequences for their job or personal life							
14. People should have the option to choose the vaccine brand they want							
15. People should be allowed to live their life with no restrictions once vaccinated							
16. Healthcare professionals and scientists with concerns about the vaccine should have opportunities to share their opinions with the public							
17. Everybody should have equal access to the most effective and safe vaccine regardless of income, race, or immigration status							
18. There is no elite group that will achieve financial power if people are getting vaccinated							
19. There is no microchip with tracking capabilities inserted in the vaccine							
